# Exploring Music-Based Rehabilitation for Parkinsonism through Embodied Cognitive Science

**DOI:** 10.3389/fneur.2015.00217

**Published:** 2015-10-19

**Authors:** Andrea Schiavio, Eckart Altenmüller

**Affiliations:** ^1^School of Music, The Ohio State University, Columbus, OH, USA; ^2^Department of Music, The University of Sheffield, Sheffield, UK; ^3^Institute of Music Physiology and Musician’s Medicine, University of Music, Drama and Media Hannover, Hannover, Germany

**Keywords:** embodiment, music therapy, Parkinsonism, dynamic systems, brain plasticity, motor rehabilitation, well-being

## Abstract

Recent embodied approaches in cognitive sciences emphasize the constitutive roles of bodies and environment in driving cognitive processes. Cognition is thus seen as a distributed system based on the continuous interaction of bodies, brains, and environment. These categories, moreover, do not relate only causally, through a sequential input–output network of computations; rather, they are dynamically enfolded in each other, being mutually implemented by the concrete patterns of actions adopted by the cognitive system. However, while this claim has been widely discussed across various disciplines, its relevance and potential beneficial applications for music therapy remain largely unexplored. With this in mind, we provide here an overview of the embodied approaches to cognition, discussing their main tenets through the lenses of music therapy. In doing so, we question established methodological and theoretical paradigms and identify possible novel strategies for intervention. In particular, we refer to the music-based rehabilitative protocols adopted for Parkinson’s disease patients. Indeed, in this context, it has recently been observed that music therapy not only affects movement-related skills but that it also contributes to stabilizing physiological functions and improving socio-affective behaviors. We argue that these phenomena involve previously unconsidered aspects of cognition and (motor) behavior, which are rooted in the action-perception cycle characterizing the whole living system.

## Introduction

Over the last three millennia, across different times, places, and cultures, music making, and music listening have been often associated with medicine (1), meditation (2), and well-being (3), serving a variety of functions deeply intermingled with everyday-life and social activities (4–9). In Ancient Greece, for example, human musical behaviors were not considered as contemplative or abstract practices, but were rather actively employed for education, religious ceremonies, celebrations, and, indeed, medical treatments (10, 11). More systematic therapeutic interventions involving music emerged after the Second World War – for example to help ex-soldiers or injured civilians recovering from stress and other related conditions (12, 13). Rehabilitative protocols adopted in this period were mostly based on models provided by the social sciences of the day – where the “cultural role of music was interpreted as an effective facilitator for therapeutic concepts of ‘wellbeing’” [(14), p. 174]. As such, the focus was on exploring how the employment of music could alleviate pain, promote emotional expression and sociality, motivate patients, and enhance self-esteem (15, 16).

From the early 2000s, with the unprecedented development of brain sciences and neuroimaging techniques, the study of music therapy shifted to a new, highly stimulating, research focus. Mirroring the same reorientation witnessed in other disciplines devoted to the study of mind,^1^ agency, and behavior, many scholars started to explore in greater details the neurological aspects related to musical activities in clinical and non-clinical contexts [see Altenmüller and Schlaug (17–19), Janata and Grafton (20), and Thaut (21)]. Within this area, a wealth of empirical evidence has showed the high degree of functional and structural plasticity of the human brain when involved in the complex demands associated with musical activity (22–26). For example, it has been demonstrated that intense Melodic Intonation Therapy (27, 28) may elicit – in patients suffering from non-fluent aphasia after left frontal lobe damage – the reactivation of inhibited language-competent brain regions in the right frontal brain networks (29–34). Additionally, other findings have confirmed the benefits of music-supported therapy in motor rehabilitation: first, studies with stroke patients revealed significant behavioral improvements in a variety of tasks related to fine motor skills (35, 36), accompanied by impressive reorganization of cortical sensorimotor networks (37, 38); second, research with Parkinson’s patients has shown that entrainment with a rhythmically rich auditory feedback may alleviate Parkinsonian gait by “increasing the excitability of spinal motor neurons via the reticulospinal pathway, thereby reducing the amount of time required for the muscles to respond to a given motor command” [McIntosh et al. (39), p. 25; see also Arias and Cudeiro (40)]. Increasingly, the clinical adoption of music-based paradigms seems to offer not only a valid non-pharmacological tool for intervention in diverse contexts [including for example pain treatment, see Bernatzky et al. (41)] but also innovative insights into the anatomy and physiology of the brain [e.g., Särkämö et al. (42)]. In general, a rich variety of empirical findings have demonstrated how musical experiences may improve the lives of patients suffering from various neurological diseases [e.g., Forsblom et al. (43) and O’Kelly et al. (44)], integrating neuroscientific and musical research in novel and fascinating ways (45, 46).

To this already fertile ground, we would like to add insights from the recent *embodied* trend, which has recently emerged in cognitive science and in philosophy of mind [e.g., Lakoff and Johnson (47), Shapiro (48), Stewart et al. (49), and Varela et al. (50)]. This framework has contributed a new and important perspective on the sciences of mind and (inter)subjectivity, with its central thesis^2^ being that cognition “depends on the kinds of experiences that come from having a body with particular perceptual and motor capacities that are inseparably linked and that together form the matrix within which memory, emotion, language, and all other aspects of life are meshed” [Thelen et al. (51), p. XX].

We argue that framing music-supported therapy within a paradigm inspired by this claim may offer useful new ways of interpreting results obtained in clinical settings, and in turn potentially improve specific protocols for interventions. Rehabilitative strategies for Parkinson’s patients, in particular, may necessitate a more unitary, holistic, view to fully appreciate the potential of music and its relevance beyond movement recovery only. This perspective aligns with recent non-reductionist trends in critical neuroscience (52–56), which emphasize the deep continuity of mind, behavior, body, brain, environment, affectivity, perception, and action; it thus contrasts with more traditional approaches where such elements are usually studied as discreet (and causally related) categories [see Colombetti (57), Kiverstein and Miller (58), and Thompson (59)].

**Figure 1:**
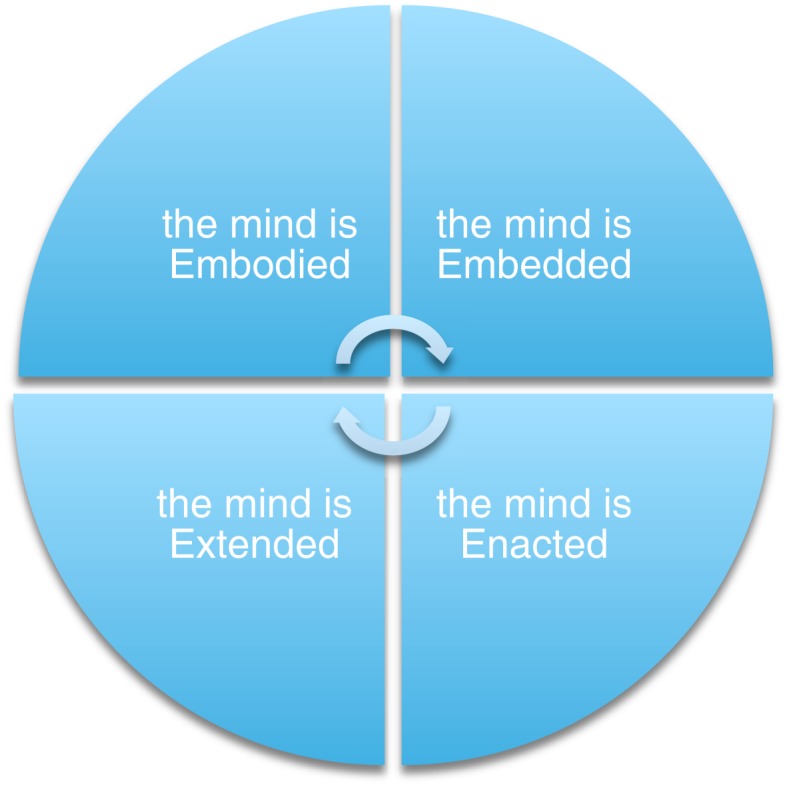
**The embodied approach in its ‘4-Es’ declinations**. As well known, however, not all versions of embodiment are extended, or enacted. Also, some of the arguments used by proponents of the extended mind thesis stand in open contrast with a truly enactive characterisation of cognition, and might eventually collapse into a functionalist account (or extended functionalism). In this paper, then, we just employ the basic points provided above and do not engage in relevant discussion.

In what follows, therefore, we discuss the need to implement insights from embodied cognitive science in research on the brain’s anatomical adaptation and for music-based motor rehabilitation. First, we introduce the embodied approach by analyzing its main tenets and its role in neuroscientific and musical contexts. Here, a brief overview of the ‘4Es’ perspective – which, as represented in Figure 1, defines cognition as Embodied, Embedded, Enactive, and Extended – is offered. Subsequently, we shift our focus to Parkinsonism, asking whether (and how) established rehabilitative protocols may benefit from the adoption of such compelling perspective. Finally, we explore possible clinical applications that the ‘4Es’ approach may inspire, showing how these may bring forth a richer understanding of the complex network of dynamical interactions between music, environment, body, brain, movement, and well-being.

## Varieties of Embodiments

The traditional ‘cognitivist’ approaches that dominated cognitive sciences for more than 50 years developed a productive research agenda that focuses principally on the role of mental representations, computations, and specialized cognitive architectures (60–62). However, analyzing how external information is acquired, processed, and represented^3^ ‘in the head’ scholars within this framework are often accused to not adequately take into consideration the body and the ecological niche in which the cognitive system is embedded (50, 63–65). Classic cognitivism, it is argued, downplays the active and adaptive engagements that unite living bodies and niche for the constitution of lived experience; and thus, in human terms, it ignores the most fundamental aspects of our being-in-the-world (66–68). Accordingly, the cognitivist framework may be seen to support a strong dichotomy between the *inner* domain of mind – functionally realized ‘in the head’ thanks to relevant domain-specific cognitive modules – and the *outer* realm of the (social and physical) ‘objective’ world, including the system’s own body (59, 69).

In contrast to this ‘orthodox’ (70) perspective, various theories of *embodied cognition* have recently emerged as new frameworks for the study of human mind. Such approaches are supported by a growing cross-disciplinary research agenda that integrates relevant contributions in theoretical biology (71), dynamic systems theory (72), linguistics (73), neurophysiology (74), phenomenological philosophy (75), cognitive science (59), and artificial intelligence (76). In general, these embodied frameworks emphasize the formative roles of bodies and environments in driving cognitive processes (59, 77–79), as well as the primacy of action over more ‘intellectual’ faculties to make sense of the world (50, 80, 81). As a consequence, cognition is now often described in terms of dynamic sensorimotor interactions between the entire body of a living system and its environment (49, 82).

While such core insights are widely endorsed by advocates of the embodied approach, its richly interdisciplinary agenda has resulted in a number of interesting formulations and interpretations (83). This growing variety of approaches to ‘embodied cognition’ has stimulated the discussion across diverse fields – promoting a highly fruitful exchange of knowledge, methodologies, and insights, while nevertheless preventing the development of a ‘standard’ framework. In talking about the *embodied approach*, therefore, we actually refer to different research programs: Embodiment, Embedment, Enactivism, and Externalism (usually labeled as ‘4Es’), which all aim to capture how bodies, brains, and environment successfully interact in real-time worldly conditions (84). These approaches hold that to understand mind we should consider how a living system *acts* in a social and physical environment (85) rather than focusing on what goes on ‘within the skull’ only. In order to get a very basic idea of the ‘4Es’ perspective, we introduce the following key points:

- Cognition does not depend solely on brain processes, but results from structures widely distributed across the whole body of a living system (the mind is *embodied*).
- Cognition arises from interactions with the (social and physical) environment; it is actively immersed in the world (the mind is *embedded*).
- Cognition can reach beyond the boundaries of skull and skin, integrating resources internal and external to the animal (the mind is *extended*).
- Cognition consists of embedded and embodied forms of interactions between a self-organized living system and its environment. Through this dynamic interplay, the creature enacts (or brings forth), its own domain of meaning (the mind is *enacted*).

It is beyond the scope of this article to discuss each of the ‘4Es’ approaches in detail; thus we will draw from them selectively – adopting both overlapping principles and distinctive insights (in particular from the *enactive* view) when necessary. While the debate over these perspectives is still heated in philosophy of mind and cognitive science, the embodied paradigm (in its four ‘E’ instantiations) has received little discussion in the context of music-based rehabilitative paradigms. But before we focus on how specific therapeutic settings may integrate existing methodological and theoretical models with insights from the embodied perspectives (mainly with regard to Parkinson’s treatment), it will be necessary to consider three basic principles associated with embodied cognition and analyze their role in neuroscience (86–88) and music cognition (89–94).

## Tracking Down Embodiment

Although the ‘4Es’ define different research agendas, they all maintain that “cognition is embodied” as their starting assumption^4^ [see Hanna and Maiese (96) and Ward and Stapleton (97)]. But what does it really mean? Broadly speaking, it is important to understand *embodiment* not as a given category that may facilitate certain aspects of perceptual and cognitive activity, or as a label to attach whenever bodily aspects are somehow involved in certain cognitive tasks. Rather, ‘embodiment’ should be intended as the pre-requisite of an agent’s being-in-the-world (98, 99). Listening to music, thinking of a good life event, feeling sad, sharing a drink with a friend, and every other possible activity we may have experience of, are all conceivable only through our *living* and *lived* body (67, 100, 101). As Di Paolo and colleagues argue: “to say that cognition is embodied is to express a tautology – it simply cannot but be embodied” [Di Paolo et al. (102), p. 42]. This passage is best understood when considering what Varela et al. (50) define as the “three dimensions of embodiment”: bodily self-regulation, sensorimotor coupling, and intersubjective interaction. Analyzed by several contributors [e.g., Thompson (59)], these insights offer a thorough perspective on the embodied view, emphasizing the explanatory power of moving beyond cognitivism across different levels of analysis. Importantly, as we will see, these “three dimensions” are extremely relevant for our discussion on music-based therapy for Parkinson’s disease (PD) patients, which we offer below.

### Bodily Self-Regulation

*Bodily self-regulation* concerns the way an agent’s biological structure contributes in regulating, modifying, and controlling its homeodynamic requirements. These processes of metabolic autonomy ensure that the agent is alive and that it maintains a stable interaction with the world. Importantly, as reported by Colombetti (103), there is no ‘self’ in self-organizing organisms: no ‘message’ is exchanged in hierarchical fashion between different independent levels via top-down or bottom-up pathways (104). Instead, the chemical, thermodynamic, and metabolic activity of the system’s sub-networks participates as a whole in maintaining the system’s homeostatic adaptivity. The process leading to adaptive stability, in which the living system (i.e., a unicellular organism, a mammalian, etc.) strives to maintain its autonomous identity, is realized through self-producing all that is needed for its maintenance (105, 106). The process, in other words, is not led by a ‘ghost in the machine,’ but rather by homeostasis (103). Consider the role of emotions, for example: seen as self-regulative processes (107, 108), they emerge within the dynamical interaction of a number of neural and extra-neural components, and not simply via an input–output sequential chain of events (57). Indeed, although defining an operationally closed network,^5^ the self-regulating processes aimed at keeping the agent’s conservation as auto-sufficient, do establish a meaningful dialectic with the environment: “whence the intriguing paradoxicality proper to an autonomous identity: the living system must distinguish itself from its environment, while at the same time maintaining its coupling; this linkage cannot be detached since it is against this very environment from which the organism arises, comes forth” [Varela (109), p. 85]. By this view, all living systems are “self-organizing thermodynamic systems with emergent truly global or inherently dominating intrinsic structure, and not mere mechanisms like a can-opener or a digital computer” [Hanna and Maiese (96), p. 20]. The integrity of self-regulative processes always involves world, body, and brain at multiple levels and time-scales (110). With regard to human musicality, these insights have been recently explored by research on bodily self-regulation in joint improvisation (111) and by the development of an enactive theory of musical emotions (Schiavio et al., under review).

### Sensorimotor Coupling

The second dimension of embodiment is *sensorimotor coupling*, which may refer to (i) the integration of sensorial and motor information occurring in the human brain (112), and (ii) the embodied forms of mutual determination established by organism and environment (113). While (i) and (ii) should always be considered as mutually dependent [(114), and see discussion in the Section “Intersubjective Interaction”], for reasons of simplification we now briefly treat them separately.

Perceptual processes, traditionally, are identified with a unidirectional stream of data from the world ‘out there’ that is retrieved, codified, and represented ‘in the head,’ eventually leading to a behavioral output (movement) (115). This process is putatively made possible by an exchange of information proceeding from the associative cortex to the agranular frontal cortex – where information is integrated with more sophisticated (i.e., decision making) aspects of intelligence. Modern neuroscience, however, is well aware of the limitations of this traditional model. Consider, for example, the highly complex cytoarchitectonic organization of the frontal lobe’s motor cortex: as Gallese (115) notes, a number of anatomical and neurophysiological findings have revealed a rich variety of anatomo-functional areas, each endowed with specific functional properties and related to each other forming distinct cortico-cortical circuits (116). This means that each of these parieto-premotor circuits continuously participates in integrating sensorial and motor information, contributing in redefining the role of the motor cortex – from a mere ‘muscle controller’ to a much more complicated system (74). Here, within the inferior frontal gyrus, the lower part of the precentral gyrus and the temporal, occipital, and parietal visual areas (117) the existence of a so-called ‘mirror’ system (116, 118, 119) has been posited to indicate a set of bimodal and trimodal neurons, which are elicited not only when doing a given action but also when observing (and/or hearing, in the case of trimodal neurons) another individual performing the same action^6^ (120, 121). Thus, it is argued that in the brain, perception and action are not separated entities somehow encapsulated in autonomous and independent modules. Rather, they are always mutually integrated through a complex web of sensorimotor connectivity, involving anticipatory mechanisms that enable the system to respond adequately to the demands of the environment (122, 123).

In league with this discussion, a number of empirical findings report the activation of neural circuits involved in motor activity and the planning of motor sequences during listening tasks (22, 124). In a well-known PET study, Halpern and Zatorre (125) demonstrated that when musicians listen to or imagine music, blood-flow significantly increases in the right supplementary motor area (SMA), a region which is implicated in motor control [see also Kristeva et al. (126)]. As Rodger et al. (127) comments, the involvement of SMA and other brain areas – i.e., basal ganglia, and cerebellum – in similar tasks (128, 129) is usually seen to support “hypotheses about the induction of a sense of beat or pulse in the listener” [Iyer (130), p. 392]. To this, we add that these findings also reflect more generally the ecological situatedness of the whole organism: listening to music involves an active, skillful, sensorimotor, exercise, which is intrinsically determined by the *sensorimotor expertise* (in terms of motor vocabulary of musical actions, for example) of the musical animal – i.e., its personal capacity to co-constitute (and act in) its niche, through the establishment of a repertoire of meaningful relationships by which it maintains its autonomous identity or a ‘point of view’ (131–134). This resonates closely with a main principle of the ‘enactive approach,’ namely, the idea that perception and action are radically entwined extraneurally in non-linear terms – and that this forms the basis for our being-in-the world (59). Put simply, from this perspective, it is not only the brain that is exposed to musical feedback. Rather, the entire living system – with his or her listening biography, body, affectivity, and history of structural couplings with the (sonic and cultural) environment – *participates* as a whole in musical experience (46, 93, 127, 135–138). We will further develop these insights when discussing of PD treatments.

### Intersubjective Interaction

The third dimension of embodiment – *intersubjective interaction* – aims to look beyond traditional ‘mentalistic’ approaches of social cognition, which often conceive of social understanding in terms of simulation-like mechanisms^7^ or through the construction of theoretical, spectatorial, models^8^ (139–142). Embodied – enactive in particular – approaches to interactivity, instead, define the processes of mutual interactions and coordination as *self-regulative* and *sensorimotor* networks (143–146). These networks are based on recursive patterns of action and perception mutually shaping each other dynamically (147). Consider two (or more) individuals playing together: no matter how much they rehearsed jointly or how many times they played the same piece, there will always be a sense in which each performance is different from one another, as even one brief ‘crescendo’ by a musician (or a particular environmental setting, or audience, etc.) will affect the other and the overall performance in real time [(138, 148, 149), p. 40]. As cognition is a process that occurs in a domain of interactions, it is realized through the *biological morphology* of the body and its dynamical and *sensorimotor interplay* with the others, where these aspects represent different *typologies* of embodiment and not separated domains (150). The body is not a rigid and fixed object, but rather a flexible entity that acts in (it modifies and responds to) the world (151); it is an “imprint of social engagement” (ibid.). The *living* and *lived* body is what allows the meaningful interactions with its environment; it is, as we stated above, the pre-condition for being-in-the-world (67, 152). The brain, accordingly, can be seen as ‘participating’ in the action rather than ‘controlling’ it^9^ (122). If cognition is realized in the domain of the system’s meaningful and embodied *interactivity*, it is not ‘located’ in any traditional sense; rather it bypasses the notions of ‘internal’ and ‘external’ (95, 114).

The notion of embodiment, therefore, encompasses all the processes connected to living subjectivity, shaping, and being shaped by the environment in which an agent is embedded. This does not only entail ‘basic’ processes – such as perception or interaction: there is growing agreement across a variety of domains in highlighting the body’s crucial role for high-level skills – such as problem solving and reasoning (153). Along these lines, it has been demonstrated that visual and rhythmic perception are shaped by looking and by body movements in both infants and adults (154–157), that motor experience facilitates memory for musical excerpts (158), and that walking is crucial for an infant cognitive development (72). Put simply, if we reduce mental life to the activity of the brain and the central nervous system, we may lose an important chance to understand the organism as ecologically situated – where bodies are not reduced to representations in the somatosensory cortex but are instead seen as constitutive category of the system’s being-in-the world. Embodied theories entail both “micro phenomena within the body, for example, the physiology of sight, the biochemistry of muscle cell contraction, [and] macro phenomena, for example, the evolution of ecosystems” [Krieger (159), p. 351]. While the relevance of these insights is recognized by different authors in the context of cognitive science and philosophy of mind, it remains partially unexplored within other domains. In the Section “A Network of Non-linear Interactions,” thus, we will consider the challenge posited by the embodied approaches more in detail, discussing how they may help us reconsider the ways in which we look at brain science.

## A Network of Non-Linear Interactions

In the last few decades, a growing number of researchers became interested not only in analyzing the cognitive operations in play while performing a musically relevant task, but also in understanding how are these operations associated with particular (networks of) brain regions^10^ (161, 162). Although most neuroscientific research has moved from functional *segregation* to functional *integration* [see Friston (163, 164)], and a number of brain scientists expressed doubts toward both neural localization and models based on a mind-brain identity as legitimate explanatory tools [see Bennett and Hacker (165) and Fuchs (166)], the tendency to look for ‘neural correlates of music processing’ nevertheless remains within musical research. As Peretz and Coltheart admit, “musical abilities are […] studied as part of a distinct mental module with its own procedures and knowledge bases that are associated with dedicated and separate neural substrates” (2003, p. 688) (167). This view of music as functionally autonomous seems to contrast with a vast range of findings in the literature, which highlight the multimodal and plastic nature of brain processing mechanisms and the widely distributed neural networks in both hemispheres this involves (168, 169). “Brain anatomy reveals that brain regions are interconnected in a rich and dense pattern, both locally and in terms of long-range connections” [Pessoa (170), p. 198]. To put it in a different way, anatomical segregations of musical functions seem to disregard the role of overlapping cortical regions and interindividual differences in brain substrates (171), as well as the observed evidence of ontogenetically developed – and rapidly adaptive – cerebral networks (24, 72). Neurons themselves display dynamical properties: there is no simple mapping from neural activity to behavior as what the neurons code depend on various time and contexts (172). Cross-sectional approaches to the study of the brain, thus, may downplay the developmental and ecological aspects shaping the living being-environment relationship (87). The brain is dynamical, self-organizing,^11^ and massively distributed (104, 174): it mediates and enables the non-linear^12^ and reciprocal interactions between the body and the world. Information, by this view, is not passively retrieved from the ‘outer word’ but rather *enacted* through the meaningful and sensorimotor activity of the organism^13^ (50).

Thus, because both genetic and ecological factors influence the development of neuronal networks (177–180) a number of scholars have found it necessary to look beyond brain reductionism (59, 88, 171, 181) and integrate traditional neuroscientific research with the study of a wider organism-world nexus (50, 57, 182). For example, recent work by Kiverstein and Miller (58), and Pessoa (170), shows how ‘structure–function’ mappings are best understood in terms of dynamical sub-components of a larger network, where a given function is highly context dependent and may vary over time in its dynamical interplay with the environment, which offers the animal various possibilities for actions according to its degree of complexity (183). It is worth noticing that the insistence on large-scale dynamic networks resonates closely with the view that sees cognition as belonging to a ‘relational domain’ (184), in which the living system acts in ways that are relevant to sustaining itself under precarious conditions. To understand the global behavior of a living organism, then, we need to do more than simply analyze one of its sub-components (i.e., the pathways underlying autonomic and muscular responses to music), as none of the system’s part controls and defines the system by itself (185). The relation between biological organization and cognitive functions is thus best understood as ‘circular,’ rather than ‘linearly causal’ (71). This is to say that an *embodied view* on human musicality – and human cognition more generally – replaces the classic input/output framework with a non-linear perturbation/response distinction, in which the brain does play a very important part, but is not the sole factor involved.

By understanding cognitive processes as widely distributed across the entire body of the animal, and into its niche, the embodied approach goes beyond brain reductionism and provides a welcome alternative to classic computational frameworks (50). In what remains, we apply these insights to clinical research, arguing that an embodied perspective may help us address some of the challenges that emerge within this context in new ways. Focusing on music-based rehabilitative paradigms for PD patients, we explore the possibility that music may not just act ‘externally’ – somehow causing relevant behavioral responses – but rather that involves the agents’ whole embodied being-in-the-world in active engagement; that it becomes a part of the network of non-linear interactions that characterizes the brain-body-world nexus (182). In doing so we hope to offer new insights into some aspects of PD treatment, and thus stimulate discussion on the interpretation and development of new approaches to rehabilitation.

## Parkinsonism and the Embodied Mind

Parkinson’s disease is a degenerative disorder associated with the progressive loss of the nigrostriatal dopaminergic neurons in the Basal Ganglia, which triggers functional changes in the same cortical network (186, 187). Non-motor symptoms are frequently the first signs and affect sense of smell and sleep regulation. Histologically, a classic mark of PD is represented by the presence of fibrillar aggregates of proteins called ‘lewy bodies,’ which displace other internal components of the remaining neurons in the midbrain, but also in the brain stem, the olfactory bulb and – at later stages – the cerebral cortex (188). The severe loss of dopaminergic cell activity in the midbrain results in hypokinetic disorders such as *akinesia* (the inability in initiating a movement), *bradykinesia* (slowness of movements) or *freezing* (impossibility to move in any direction) [see Berardelli et al. (189) and Grabli et al. (190)]. Usually, one of the first symptoms associated with PD is represented by an involuntary 4–5 Hz resting movement (191); clinical observations suggest that this *tremor* may disappear in voluntary actions, but can worsen with ambulation and with ‘Froment’s maneuver’ (contralateral motor activity) (192). As the condition progresses, tremor is often accompanied with muscle *rigidity*, which leads to resistance of externally imposed joint movements (193). While states of relaxation may help, patients who are asked to move the contralateral limb often exhibit – like with tremor – an aggravation of the symptom (194, 195). Other typical motor deficits (often, but not always, emerging in later-stage PD) are *postural instability* and *gait disorders*, which result in an increased risk of falls (a predictor of mortality) and in turn critically challenge independent living habits and quality of life more generally (196, 197). Symptomatically, treatments with dopaminergic agonists or deep-brain stimulation have been demonstrated to be partially effective with many of these motor disorders (198–200), and are thus often integrated with non-invasive techniques based on music and rhythmic engagement (201, 202).

### Extending the Loop

A growing wealth of evidence shows how the contribution of music-based interventions is important for improving symptoms such as Parkinsonian gait (203–205). By matching their walking to the musical beat, or to a metronome, PD patients normally exhibit considerable benefit in terms of velocity, cadence, and stride length (206, 207). Interestingly, auditory cues for this kind of treatment display advantages when compared to visual, somatosensory, or combined cues: not only is reaction time to auditory cues shorter when compared to visual and tactile ones, but ‘periodicity’ is also best captured in sonic contexts rather than through other sensory systems^14^ (210, 211). Indeed ‘timing’ and ‘periodicity’ are fundamental aspects for gait, ensuring adequate consistency in pace and stability. As basal ganglia-cortical circuitry is typically involved in time-related processes – with a series of structures depending on dopaminergic innervation – its malfunctioning in PD has a significant impact on timing and motor synchronization (152, 212–214).

This is not to say, however, that ‘timing’ can be understood as a high-level cognitive ability that is functionally autonomous and encapsulated in the brain. First, besides the basal ganglia, it is likely that other cortical regions contribute in timing processing, thus constituting a distributed network that includes the cerebellum, SMA, pre-SMA, inferior parietal cortex, and premotor cortex (215–217). Moreover, the basal ganglia itself is involved in the selection and inhibition of motor processes (218), highlighting the deep connectivity of categories such as action, body, and ‘timing.’ Second, such connectivity implies that we cannot understand what ‘timing’ and ‘periodicity’ entail if we do not look beyond the boundaries of skull and skin to consider how the whole embodied agent *participates* in gait. Walking and synchronizing with a beat do not happen ‘in the head’; they occur in the concrete sensorimotor dynamics of the world in which we are embedded, a world that is meaningful and rich of affordative structures ready to be acted upon. Music offers such affordances (219) according to the history of structural couplings between music users and sonic environment(s) (91, 92, 94, 133, 137, 138, 220, 221). We shall return to this point in the Section “Beyond Motor Recovery.” What we want to stress, here, is that the organism’s body,^15^ in its ‘motor resonance’ with the beat, enables the fluidity of the gait’s ‘kinetic melodies’ in a continuous dynamical process of action and perception. This means that ‘timing processes’ – as subcomponents of the distributed network enabling gait – involve the entire body, and the world, literally *extending* beyond skull and skin. Thus musical rhythm offers a new pathway to enact self-organization through sensorimotor coupling by compensating for the malfunctioning of one of the system’s sub-networks. The hyperactivity recorded in the cerebellum and in the pre-SMA at the preclinical stage (223–225) seems to confirm these network’s self-organizing properties, which tend to develop other processes to counterbalance the impaired sensorimotor circle dynamically. As pre-SMA will eventually become hypoactive, left and right cerebellum and contralateral motor cortex have been observed as hyperactive also at later stages (226). Moreover, the compensatory mechanisms emerging in PD’s pre-clinical and clinical stages show that self-organization also occurs on an ecological scale, integrating resources internal and external to the patient. Therefore, positing a single brain-body-world nexus – instead of the classic model based on the separation between internal (brain-bound) and external (worldly) domains – may help us better capture and model the ways in which the reorganization of the nexus’ sub-networks unfolds in terms of dynamical and continuous interplay with the environment (175, 227, 228).

This process of wordly self-regulation, in which patients aim to recalibrate their sensorimotor engagement with the world, should also comprehend the ‘social dimension’ of embodiment, as the world involves other agents by definition. Stressing the importance of social interactions in a patient’s being-in-the-world, it would be thus interesting to see how PD patient would respond to the so-called ‘perceptual crossing paradigm,’ which has been recently developed to study real-time situations in non-individualistic terms (229). Its simple methodology, which involves only “two subjects, a one-dimensional space, and a yes/no answer” (230), makes it particularly suitable for clinical contexts, and may illuminate on how PD affects the patient’s capacity to interact with others. In the original experiment, as reported by Auvray et al. (229), pairs of blindfolded subjects in different rooms are asked to interact with each others in a computer-generated space. Participants are asked to move a cursor in this virtual space, clicking a mouse button when they perceive the presence of another participant. But since subjects are blindfolded, they only receive a tactile stimulation on the free hand when their avatar crosses an object in the one-dimensional space. There are three different types of objects to be encountered: (i) the moving avatar of another participant, (ii) an object placed in a fixed location by the experimenters, and (iii) the moving ‘shadow image’ of the partner’s avatar, that is an object that reproduces at a displaced distance the same movements of (i). The only difference between (i) and (iii), thus, is that with (i) a dyadic interaction is possible. As Froese and Di Paolo comment:

The two mobile objects exhibit exactly the same movement, but only an overlap of the receptor fields of both participants gives rise to mutual sensory stimulation. Note that the difference between these three types of objects cannot be directly provided by the sensors, which in all cases can only produce a binary, all-or-nothing response depending on whether something is overlapping their particular receptor field or not. Thus, if the participants are to be successful at distinguishing which of the objects is the other agent’s receptor field, they must accordingly rely on differences in the kinds of interactions that these objects afford. The results of the psychological study show that, at least under the minimalist conditions of this experiment, the successful recognition of an ongoing interaction with another person is not only based on individual capacities. It is also based on certain properties that are intrinsic to the joint perceptual activity itself [Froese and Di Paolo (231), p. 49].

Indeed, participants displayed greater accuracy in clicking the button when meeting the partner’s avatar (65.9% of the clicks ± SD of 13.9) when compared to meeting the shadow image (23.0 ± 10.4%) or the static object (11.0 ± 8.9%) [see again Auvray and Rohde (230)]. In the case of PD patients, we predict a significant decrease in correct answers, as their ability to interact with the world might be partially compromised by the condition. The results, however, might be improved by exposure to *motorically familiar* musical cues. Indeed, hypothesizing that a malfunctioning sensorimotor coupling with the world makes the body an ‘obstacle’ for the living system’s being-in-the-world (232), listening to music one can play may help to re-establish the correct sensorimotor loop with the environment through a ‘motor resonance’ enabled by the mirror mechanism. In the Section “Beyond Motor Recovery,” we will try to describe how such hypothesis could be tested adequately, generating predictions that involve the whole living system in its dynamic interplay with the environment – and not only movements’ rehabilitation.

### Beyond Motor Recovery

It is likely that the ‘motor resonance’ in play during music based motor rehabilitation involves the mirror mechanism mentioned above, as it does not seem to be significantly altered by PD (233). The activation of sensorimotor networks during music listening is well known (234, 235) – with musicians and subjects who have a practical knowledge of the complex order actions required to obtain a particular music showing stronger activations in the front-parietal-temporal network (132, 236). While the interpretation of such work is still a subject of controversy (237–239), it may nevertheless be argued that a ‘motor vocabulary’ of musical actions is formed when learning music. However, the firing of the neurons that might constitute such a ‘vocabulary’ (or ‘repertoire’) during listening tasks need not be understood in terms of ‘information processing.’ Rather it may be seen as allowing the system to *prepare for action*, possibly underpinning “a non-articulated immediate perception of the other person’s intentional actions” [Gallagher (240), p. 541; see also Gallagher (147)]. As preparation for action is indeed an important component of intersubjective contexts – both phylogenetically and ontogenetically – mirror neuron theories may help us understand some other aspects of PD rehabilitative strategies. For example, they can explain why simple rhythmic excerpts or metronomic beats are widely and successfully adopted in this type of clinical research: almost everyone possesses (i.e., acquires through development) the motor expertise necessary to produce a repetitive beat. In this sense, the relationship between music and living systems is literally shaped by the history of structural sensorimotor coupling between them. Thus, the (therapeutic) compensatory mechanisms resulting from musical exposure appear to work when listeners-patients possess the adequate (meaningful) motor expertise relevant to re-enact the goal-directed actions afforded by the auditory cues.

A way to test this hypothesis in PD-related contexts might involve *familiarizing*^16^ subjects at an early clinical stage with musical stimuli that are more complicated than a simple beat-pulse, and then observing at a later stage of the rehabilitation whether the same stimuli are more beneficial for gait (and – as it will emerge later – for more general improvement) when compared to standard simple beat or to unfamiliar music. This is to say that patients are not passive ‘responders’; rather they actively ‘enact’ their own meaningful vocabulary of musical actions during music-based interventions, bringing forth their ‘autonomous identity.’ Increased familiarity with music-making in both individual and collective settings could foster the development of intersubjective rehabilitative contexts, where the interactivity of patients may generate more efficient results – increasing demands in sensorimotor integration. Indeed, this approach might be taken further to involve patients in music improvisation and the co-creation of musically relevant stimuli. Put simply, we suggest that by encouraging patients to develop more complex rhythmic-musical understandings, which they then develop and apply in the course of their treatment, new clinical possibilities may emerge that involve patients more comprehensively across the range of their being. In this way, treatment that involves increasingly adaptive and creative interactions with the environment (musical stimuli and other patients), may foster ways of being-in-the-world that lead to improved self-regulation, as well as a renewed, and much needed, sense of agency. Along these lines, the use of more sophisticated musical cues, and more intersubjective settings, might also lead to beneficial results beyond the motor domain. This is important, if we consider that a cascade of other non-somatic symptoms^17^ often accompanies the motor dysfunctions described in the Section “Extending the loop”: half of PD patients, for example, are reported to develop depression (241). But how could embodied theories say something about depression? And how could music-based motor rehabilitation help?

Relevant applications in clinical settings stemming from embodied theories have been recently explored within neuropsychiatric and psychopathological research – for example in schizophrenia (242) and depression (232). Research on the latter, in particular, suggests that depressive patients display similar symptoms to those of PD patients, including slow gait and reduced stride length (243–245). Indeed, like the PD sufferer, the depressive subject experiences a loss in their dynamical relation with the world and “cannot retain a position outside of her body” [Fuchs (53), pp. 99–100]. This is important when considering that, as Kyselo and Di Paolo (246) report, without the bodily power of action (for example in case of global paralysis) a subject may also suffer a decrease of cognitive activities such as imagery and goal-directed thinking [see Kübler and Birbaumer (247)]. Consider the following passage, where Fuchs and Schlimme (232) describe depressive melancholia as a case of ‘hyperembodiment.’ The authors argue that the process of becoming separated from the living system’s peripersonal space results from psychomotor inhibition (as in PD) and a loss of the conative dimension of the body – its “affective and appetitive directedness”:

Normally, it is this [conative] dimension that opens up the peripersonal space as a realm of possibilities, “affordances” and goals for action. In depressive patients, however, drive and impulse, appetite and libido are reduced or lost, no more disclosing potential sources of pleasure and satisfaction. Confined to the present state of bodily restriction, depressive patients cannot transcend their body any more. The open horizon of possible experiences shrinks into a locked atmosphere, in which everything becomes permeated by a sense of lost possibilities. With growing inhibition, sensory–motor space is restricted to the nearest environment, culminating in depressive stupor. Thus, melancholia may be described as a reification or “corporealization” of the lived body, or as a “hyperembodiment” [Fuchs and Schlimme (232), pp. 572–573].

By this view, therapeutic interventions can be seen as an attempt to re-establish the functioning of the agent-environment system as a whole. Integrating standard rehabilitative settings for motor recovery in PD patients with more complex stimuli and activities, in early and later clinical stages, may lead to more beneficial results in terms of reshaping the motor resonance with the environment. These results are not limited to the motor domain, but may cover also non-somatic aspects of the pathology, as in the case of depression. Art-based therapies in general, and music therapies in particular, have been widely employed in the treatment of unipolar depression (248, 249), leading to encouraging results. An example comes from dance therapies, which have been proven effective in improving physical fitness and well-being more generally (250–252).

Acting upon the conative dimension of the sensorimotor coupling with the world, we argue that developing more meaningful musical environments could help in stabilizing the patients’ embodied being-in-the-world (in a better fashion than with unfamiliar music or rhythmical beats only) by engaging the interactivity of the entire living system. The mechanisms underlying this are to be found in the neural compensatory mechanisms elicited by musical participation, and by the active engagement of the body in the concrete dynamics of action (253). Without positing a clear input–output relation between music and patient, an embodied approach to PD treatment with music emphasizes the self-regulatory aspects of brains and bodies, conceived as unities inseparable from their niche. Also, it conceives PD as a disturbance of the subjective sensorimotor skills to engage with the world, rather than solely a neurological pathology. Music, here, does not only influence the excitability of given neurons, but offers a new affordative space to the recovering embodied agent, compensating for the malfunctioning action-perception loop that characterizes the disease. It is important to stress once again that this does not exclude affectivity but, on the contrary, highlights the conative dimension of the living body as integrative part of its perceiving, knowing, doing, and being – opening new and fascinating possibilities for health and well-being.

## Conclusion

In avoiding the twofold reductionism of anatomical specificity and information-processing generality, the embodied trend provides a considerable challenge to established theoretical frameworks concerning the nature of mind, behavior, and agency. ‘Embodiment,’ although declined differently through the ‘4Es’ described above, embraces the centrality of self-regulation, sensorimotor coupling, and intersubjective interactions for understanding the complex nature of our being-in-the-world (98, 100). We are confident this general reorientation can stimulate the development of new conceptual tools and research methods that may enhance standard rehabilitative settings within clinical contexts. In this paper we focused on how this may occur in PD research, hypothesizing that music based therapeutic settings could become even more efficient if coherently informed by theoretical models inspired by an embodied account to cognition.

Empirically, the adoption of embodied insights emphasizes the need to develop new experimental methods that are able to capture the way in which possible perturbations (i.e., a malfunction of a given sub-network) destabilize the whole brain-body-world system. Strategies for intervention, by this light, should not focus only on the isolated symptom thought to provoke a desired behavioral output (104). Rather, an embodied approach to motor rehabilitation should also consider, for example, conative, agentic, creative, and intersubjective dimensions as fundamental for the treatment of the patient – perhaps manipulating the degree of mutual interaction and affective experience according to the motor knowledge of the patient. Indeed, a musical stimulus (beyond a mere beat) is not only a ‘timekeeper,’ but also an actual *tool for cognition*, a meaningful event that affords a variety of self-regulative, interactive, and sensorimotor processes depending of the agent-music interaction’s degree of complexity. With this in mind, and drawing on insights from research on mirror neurons, we hypothesized that PD patients might benefit from familiarization phases with more complex stimuli beginning in the early stages of the disease. The compensatory mechanisms in play during exposure to musical rhythms might then be more widely effective in the recovery of other (i.e., depressive) symptoms. This is just one example of how embodied approaches may define a broader approach to the study of PD rehabilitation, and why it necessitates further discussion and testing.

Overall, what we want to emphasize is that, theoretically, this kind of non-reductionist approach may be fundamental in rethinking many taken-for-granted assumptions concerning health and well-being, neuroscience, and music research. While the operational domain of the system’s internal (e.g., brain) states is certainly fundamental to the interactive processes of such interactions, these internal processes alone cannot be identified with ‘cognition’: to do so “is to confuse levels of discourse or to make a category mistake (neurons do not think and feel; people and animals do)” [Thompson and Stapleton (95), p. 27]. In other words, the processes allowing the system to maintain itself as autonomous are realized in the sensorimotor, dynamic, affective, interplay between bodies (including brains) and environments. These processes, as a whole, are not strictly speaking “neural,” but rather define a non-linear network constituted by *both* neural and extra-neural interactive sub-networks (50).

Consider, for example, research in music psychology: while it is diverse and interdisciplinary, incorporating both ‘subjective’ (i.e., introspective, qualitative) and ‘objective’ (i.e., quantitative) methodologies (254), a common tacit assumption in the field is that (musical) experience is inner, computationally implemented, and reducible to neural activation. Embodied approaches challenge this perspective, showing that human musicality is deeply *embodied* (being constantly implemented by sensorimotor feedbacks and real-time bodily activities), *embedded* (as it is always situated in specific sociocultural niche), *enacted* (relying on the history of structural couplings between musical agents and musical environment) and *extended* (as no clear boundaries between internal and external resources exist in driving cognitive processes). And likewise, embodied perspectives represent a call for a new kind of integrative and non-reductionist music therapy – one that explores the possibilities of human musicality from diverse perspectives; and that may transform motor rehabilitation into a participatory activity where motion, emotion, listening biographies, and neural networks are all involved in a complex recursive interplay (255).

Data on brain and behavioral activities has contributed greatly to new perspectives on the audio-visuo-motor integration underlying musical experience. We wish to stimulate researchers to integrate this body of knowledge with a critical analysis of the theoretical models underlying rehabilitative contexts (i.e., information-processing). Thus, by moving beyond traditional input-output and stimulus-response paradigms, our approach identifies large-scale networks (inside and outside the skull) as a solid alternative to reductionist approaches – highlighting the explanatory role of embodied perspectives in describing how an autonomous system develops, stabilizes, and transforms according to the reciprocal influences of local and global factors. In this way, hypokinesia, tremor, rigidity – but also depression, and other non-somatic symptoms – may be understood in a new light: as affecting the patient’s *being-in-the-world* in a way that requires an recalibration of the *whole* brain-body-world network. In this way, the development of richer, embodied approaches to music intervention for PD (and other disorders) not only offers possibilities for improving the general quality of life of patients, it may also help us better understand how therapeutic recalibration occurs providing additional insights for clinical, musical, and neuroscientific, research.

## Conflict of Interest Statement

The authors declare that the research was conducted in the absence of any commercial or financial relationships that could be construed as a potential conflict of interest.

## Footnotes

^1^‘Mind’ and ‘cognition’ are used as synonyms in this paper.

^2^As we will see, this main claim inspired the development of different research programs.

^3^Representational structures are usually defined by computational procedures that operate in light of (are realised by) functionally autonomous cortical regions.

^4^Although there is certainly a continuity among these perspectives, it should be noted that some of the arguments used by proponents of the extended mind thesis stand in open contrast with an enactive characterisation of cognition, and might eventually collapse into a functionalist-like framework, where computations and representation would still play a key role in driving cognitive processes, even if coupled with external resources [see Thompson and Stapleton (95)].

^5^Indeed, the internal self-regulative processes are theoretically sufficient to maintain the agent’s homeostatic stability.

^6^As it is well known, these “mirror neurons” do not code the precise movement performed by another individual but, rather, the goal of the given action. As long as the goal of a given action is different, these neurons are selective enough to distinguish between different kinematic schemas (74).

^7^The theory holds that understanding the behaviours and the beliefs of others is a matter of simulating them internally with my own cognitive system – “as if” I was behaving that way or possessing that belief.

^8^Accordingly, people manage to make sense of the others by developing a “common-sense” theory of mind consisting of sets of laws connecting inner states to external stimuli, other inner states, or behaviours.

^9^As Di Paolo and colleagues insist: “Embodiment means that mind is inherent in the active, worldful body, that the body is not a puppet controlled by the brain but a whole animate system with many autonomous layers of self-coordination and self-organization and various degrees of openness to the world that create its sense-making activity” [Di Paolo et al. (102), p. 42].

^10^From the focus on the theoretical level between physical implementation and behavioural output (as in classical cognitivism), the exploration of the brain’s functional neuroanatomy led a number of scholars to maintain that different brain regions represent different musical functions, such that neural tissue at a specific location govern given cognitive features [e.g., Tan et al. (160)].

^11^“Systems are self-organized when there is a reciprocal relationship among local areas and behavior at the global state of the system” (173).

^12^These interactions are “non-linear” because in a system of reciprocal continuity there is no clear “input” or “output” (59, 104).

^13^Consider, for example, the nervous system: its inner states are always stimulated by the environment, but not influenced causally through an input-output relation; “the nervous system does not receive information. It rather creates a world by defining which configurations of the milieu are stimuli” [Weber (106), p. 15]. Functional localisation, reductionism, and ‘internalist’ frameworks are unable to capture these aspects by definition, as self-organisation occurs at a larger ecological scale (175, 176).

^14^To clarify this point, consider the apparent universality and spontaneity of sensorimotor synchronisation and impulse to move with music: evolutionary, this phenomenon may have promoted coordination and emotional communication (208, 209), contributing in strengthening the link between musical behaviours and wellbeing in intersubjective contexts.

^15^It is important to note that here we refer to “body” not as an objective piece of the world – the German “Körper” – but also as “Leib,” a living and lived body with its autonomous and dynamical layers of self-organizational adaptivity [see Gallagher and Zahavi (222) and Merleau-Ponty (67)].

^16^That is, developing the sensorimotor skills required to perform the musical stimuli.

^17^Other symptoms may include hallucinations, disorders of sleep and behaviour, dementia, psychosis, decrease of attention and memory, and language impairment.
